# The impact of severe COVID-19 on health-related quality of life and disability: an early follow-up perspective

**DOI:** 10.5935/0103-507X.20220008-en

**Published:** 2022

**Authors:** Liliana Cristina da Silva Ferreira Fontes, Paulo Jorge Ribeiro Costa, Joana Carolina João Fernandes, Tatiana Santos Vieira, Nuno Cruz Reis, Isabel Maria Metelo Coimbra, José Artur Osório Carvalho Paiva

**Affiliations:** 1 Department of Intensive Care Medicine, Centro Hospitalar Universitário São João - Porto, Portugal.

**Keywords:** COVID-19, Coronavirus infections, Betacoronavirus, SARS-CoV-2, Critical care, Quality of life, Disability evaluation, Patient discharge, Rehabilitation

## Abstract

**Objective:**

To assess early postdischarge health-related quality of life and disability of all survivors of critical COVID-19 admitted for more than 24 hours to na intensive care unit..

**Methods:**

Study carried out at the Intensive Care Medicine Department of *Centro Hospitalar Universitário São João* from 8^th^ October 2020 to 16^th^ February 2021. Approximately 1 month after hospital discharge, an intensive care-trained nurse performed a telephone consultation with 99 survivors already at home applying the EuroQol Five-Dimensional Five-Level questionnaire and the 12-item World Health Organization Disability Assessment Schedule 2.0.

**Results:**

The mean age of the population studied was 63 ± 12 years, and 32.5% were submitted to invasive mechanical ventilation. Their mean Simplified Acute Physiologic Score was 35 ± 14, and the Charlson Comorbidity Index was 3 ± 2. Intensive care medicine and hospital lengths of stay were 13 ± 22 and 22 ± 25 days, respectively. The mean EuroQol Visual Analog Scale was 65% (± 21), and only 35.3% had no or slight problems performing their usual activities, most having some degree of pain/discomfort and anxiety/depression. The 12-item World Health Organization Disability Assessment Schedule 2.0 showed marked impairments in terms of reassuring usual work or community activities and mobility. The use of both tools suggested that their health status was worse than their perception of it.

**Conclusion:**

This early identification of sequelae may help define flows and priorities for rehabilitation and reinsertion after critical COVID-19.

## INTRODUCTION

In recent decades, reintegration of critical illness survivors in the community with good standards of quality of life has been globally considered the most critical outcome measurement.

Advances in intensive care medicine (ICM) have resulted in a growing number of survivors after life-threatening conditions, leading to more individuals developing important long-term sequelae.^(1)^ This situation affects not only the survivors but also their families and loved ones, possibly inducing a decrease in health-related quality of life (HRQoL).^(2,3)^

In 2010, “the Society of Critical Care Medicine coined the term Post Intensive Care Syndrome (PICS) to describe new and persistent declines in physical, cognitive, and mental health functioning that follow an ICU stay and for which other causes, such as traumatic brain injury (TBI) or cerebrovascular accident (CVA), have been excluded”.^(4)^

Although no consensus is available concerning the optimal timing to start a follow-up program, the sooner patients at risk of or with post-intensive care syndrome (PICS) can be identified, the better the rehabilitation and reintegration processes can be maximized.^(5)^ Additionally, the National Institute for Health and Care Excellence (NICE) guidelines advocate a program that follows the patient in all disease phases, defending the advantages of the early detection and referencing of their needs.^(6)^

Coronavirus disease 2019 (COVID-19) survivors of critical illness are potential candidates to develop PICS, possibly related to the characteristics of the disease itself or the type of intensive care treatment needed - e.g., deep sedation and long length of stay (LOS) - which may negatively influence their quality of life.^(7,8)^

In Portugal, after a first wave of COVID-19 in March and April 2020, a devastating second wave occurred, starting in October 2020. The response to the overwhelming first wave allowed our Department of ICM to use the learning curve to better respond to the second wave - namely, by structuring a specific follow-up process after the hospital discharge of critical COVID-19 patients. The follow-up process included a nursing phone consultation approximately 1 month after hospital discharge and a medical consultation three, six and twelve months later, with the establishment of referrals and facilitators to other consultations—namely, rehabilitation, psychiatry and urology.

This study aimed to assess, approximately 1 month after hospital discharge, HRQoL and the health and disability of each patient survivor of severe COVID-19 admitted to our Department of ICM with a LOS > 24 hours and discharged home.

## METHODS

This descriptive retrospective study included all COVID-19 survivors admitted to the Department of ICM of *Centro Hospitalar Universitário São João* (CHUSJ) after 8 October 2020, with an effective hospital discharge until 1^st^ March 2021 and with an intensive care stay greater than 24 hours. This project was approved by the Ethics Committee of CHUSJ (authorization number 271/21).

The inclusion criteria were adult patients admitted to the Department of ICM because of severe acute respiratory syndrome coronavirus 2 (SARS-CoV-2) infection for a period longer than 24 hours between October 8, 2020 and February 16, 2021 and discharged from the hospital until 1^st^ March 2021.

The exclusion criteria were as follows: a LOS in the Department of ICM of less than 24 hours and discharge to be admitted to social support institutions or to other hospitals, either public or private.

In the first month after hospital discharge, an intensive care-trained nurse performed a telephone consultation with all survivors to assess HRQoL and health and disability. All consultations were performed by the same nurse, with long-term experience in intensive care follow-up, using the same scales and following the application instructions such as the Portuguese versions validated by original authors, counseling and managing problems in collaboration with the multidisciplinary team of Department of ICM follow-up.

To evaluate HRQoL and health and disability, we applied a widely used tool to evaluate the self-perception of survivors, such as EuroQol Five-Dimensional Five-Level questionnaire (EQ-5D-5L) and the 12-item World Health Organization Disability Assessment Schedule 2.0 (WHODAS 2.0),^(9,10)^ which assesses the health status and disability, developed by the World Health Organization (WHO).

The EQ-5D-5L and the EuroQol Visual Analog Scale (EQ-VAS) were applied to assess quality of life,^(11)^ and the 12 items WHODAS 2.0^(12,13)^ was used to measure health and disability.

Each question of 12 items WHODAS 2.0 is pooled into one of six different domains of disability evaluation as described by Federici et al.,^(14)^ “A 12-item version consisting of two items from each domain (Understanding and communicating, Items 3 and 6; Getting around, Items 1 and 7; Self-care, Items 8 and 9; Getting Along with People, Items 10 and 11; Life activities, Items 2 and 12; Participation in society, Items 4 and 5)”. 12 items WHODAS 2.0 allows a more objective assessment of disability.

Telephone contact was performed between 13^th^ November 2020 and 1^st^ March 2021. The conversation was performed with the patient, either directly or, if this was impossible, with the next of kin serving as an intermediate of the patient’s responses. Only in 12 cases (12.1%) did the next of kin serve as an intermediate of the patient’s responses. The information collected in the telephone nursing consultation was recorded in the patient’s electronic clinical file in the informatics system used in the department (B-ICU.CARE©). An Excel file was used by the research team to facilitate the statistical analysis. All the patients evaluated were later reassessed in a medical consultation, and this phone nurse assessment prioritized the scheduling of that consultation.

## RESULTS

**Figure 1:**
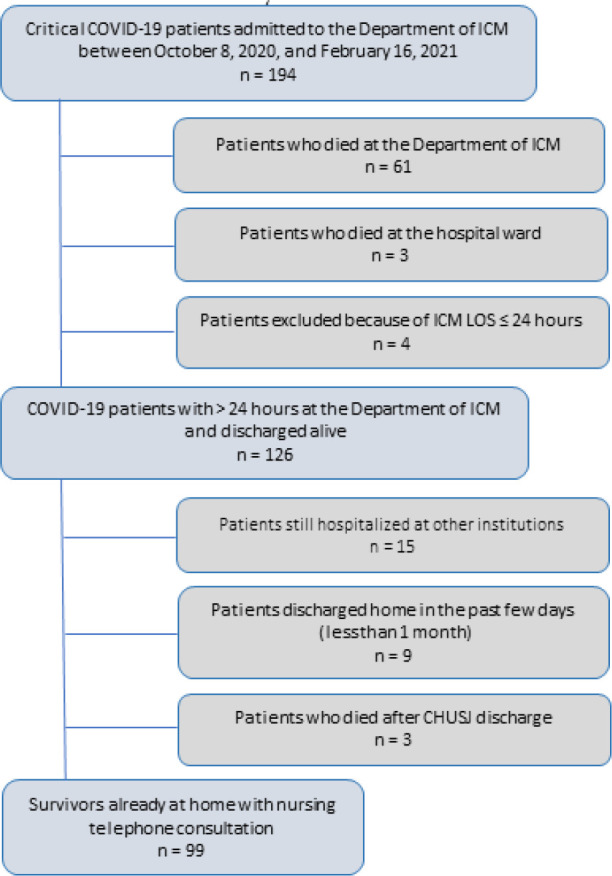
Diagram of COVID-19 critical care patients selected for the study. ICM - intensive care medicine; LOS - length of stay; CHUSJ - *Centro Hospitalar Universitário São João*.

A total of 194 critically ill COVID-19 patients were admitted between 8^th^ October 2020 and 16^th^ February 2021. Among the 126 hospital survivors who were discharged from our hospital until 1^st^ March 2021, 111 were discharged home. Ninety-nine survivors were enrolled in this study. The reason for the noninclusion of the other 12 patients is presented in figure 1, and their demographic and clinical characteristics are detailed in table 1.

**Table 1:** Characteristics of second wave critical COVID-19 survivors

Total number of nursing telephone consultations	99 (100)
Men	63 (63.6)
Age (years)	63 ± 12
Number of patients aged < 60 years	34 (34.3)
Days between hospital discharge and nurse telephone contact	20 ± 7
Days from COVID-19 symptoms onset and telephone nursing consultation	53 ± 21
Charlson Comorbidity Index	3 ± 2
SAPS II	35 ±14
TISS 28	32 ± 7
ICM LOS (days)	13 ± 22
Hospital LOS (days)	22 ± 25
Patients supported with IMV	32 (32.3)
Patients supported on NIV or HFNC not supported with IMV	63 (63.6)
Patients on ECMO	w3 (3)
Duration of IMV	24 ± 23
Duration NIV	4 ± 3
Duration of HFNC	5 ± 5
Duration of ECMO	21 ± 17

SAPS - Simplified Acute Physiology Score; TISS- therapeutic intervention scoring system; ICM - intensive care medicine; LOS - length of stay; IMV - invasive mechanical ventilation; NIV - noninvasive mechanical ventilation; HFNC - high flow nasal cannula; ECMO - extracorporeal membrane oxygenation. The results are expressed as n (%) or mean ± standard deviation.

EuroQol measurements of the health status using the EQ-5D-5L are shown in table 2. In summary, the five dimensions assessed were as follows: mobility (most of our population reported no problems walking, with 38.4%, or slight problems, with 34.3%); self-care (the majority - 63.7% - reported no problems washing or dressing themselves); usual activities (22.2% reported no problems performing their usual activities; 25.3% had moderate problems, and 30.3% could not perform those actions); pain/discomfort (43.4% reported no pain or discomfort); anxiety/depression (35.4% reported no anxiety or depression, and 32.3% reported slight anxiety or depression).

**Table 2:** EuroQol Five-Dimensional Five-Level questionnaire

Dimension	Level	n (%)
Mobility	I have no problems in walking about	38 (38.4)
	I have slight problems in walking about	34 (34.3)
	I have moderate problems in walking about	18 (18.2)
	I have severe problems in walking about	7 (7.1)
	I am unable to walk about	2 (2.0)
Self-care	I have no problems washing or dressing myself	63 (63.7)
I have slight problems washing or dressing myself	13 (13.1)
I have moderate problems washing or dressing myself	13 (13.1)
I have severe problems washing or dressing myself	4 (4.0)
I am unable to wash or dress myself	6 (6.1)
Usual activities	I have no problems doing my usual activities	22 (22.2)
I have slight problems doing my usual activities	13 (13.1)
I have moderate problems doing my usual activities	25 (25.3)
I have severe problems doing my usual activities	9 (9.1)
I am unable to do my usual activities	30 (30.3)
Pain/discomfort	I have no pain or discomfort	43 (43.4)
I have slight pain or discomfort	31 (31.4)
I have moderate pain or discomfort	22 (22.2)
I have severe pain or discomfort	2 (2.0)
I have extreme pain or discomfort	1 (1.0)
Anxiety/depression	I am not anxious or depressed	35 (35.4)
I am slightly anxious or depressed	32 (32.3)
I am moderately anxious or depressed	23 (23.2)
I am severely anxious or depressed	2 (2.0)
I am extremely anxious or depressed	7 (7.1)

The response to the question “how good or bad your health is TODAY?” on a scale from 0 to 100 (EQ-5D VAS) was a mean of 65% (± 21).

The 12 items WHODAS 2.0 assess six domains of health and disability and the data regarding our population are expressed in table 3. In summary, the different domains were as follows:

**Table 3:** World Health Organization Disability Assessment Schedule 2.0 12 item

Domain	Question	Difficulty	n (%)
Cognition	S3 - Learning a new task, for example, learning how to get to a new place?	None	20 (20.2)
Mild	2 (2.0)
Moderate	2 (2.0)
Severe	1 (1.0)
Extreme or cannot do	2 (2.0)
NA*	72 (72.8)
S6 - Concentrating on doing something for ten minutes?	None	85 (85.9)
Mild	3 (3.0)
Moderate	4 (4.0)
Severe	1 (1.0)
Extreme or cannot do	5 (5.1)
NA*	1 (1.0)
Mobility	S1 - Standing for long periods such as 30 minutes?	None	39 (39.5)
Mild	4 (4.0)
Moderate	8 (8.1)
Severe	0 (0.0)
Extreme or cannot do	24 (24.2)
NA*	24 (24.2)
S7- Walking a long distance such as a kilometer [or equivalent]?	None	17 (17.2)
Mild	8 (8.1)
Moderate	3 (3.0)
Severe	0 (0)
Extreme or cannot do	57 (57.6)
NA*	14 (14.1)
Getting along	S10 - Dealing with people you do not know?	None	76 (76.7)
Mild	3 (3.0)
Moderate	6 (6.1)
Severe	0 (0)
Extreme or cannot do	5 (5.1)
NA*	9 (9.1)
S11 - Maintaining a friendship?	None	80 (80.8)
Mild	6 (6.1)
Moderate	7 (7.1)
Severe	2 (2.0)
Extreme or cannot do	3 (3.0)
NA*	1 (1.0)
Life activities	S2 - Taking care of your household responsibilities?	None	33 (33.2)
Mild	9 (9.1)
Moderate	17 (17.2)
Severe	5 (5.1)
Extreme or cannot do	27 (27.3)
NA*	8 (8.1)
Domain	Question	Difficulty	
Life activities	S12 - Your day-to-day work/school?	None	3 (3.3)
Mild	0 (0)
Moderate	3
Severe	0 (0)
Extreme or cannot do	35 (35.4)
NA*	58 (58.6)
Participation	S4 - How much of a problem did you have joining in community activities (for example, festivities, religious or other activities) in the same way as anyone else can?	None	13 (13.1)
Mild	1 (1.0)
Moderate	1 (1.0)
Severe	0 (0)
Extreme or cannot do	57 (57.6)
NA*	27 (27.3)
S5 - How much have you been emotionally affected by your health problems?	None	11 (11.2)
Mild	13 (13.1)
Moderate	22 (22.2)
Severe	33 (33.2)
Extreme or cannot do	19 (19.3)
NA*	1 (1.0)
Domain	Question	Difficulty	n (%)
Life activities	S12 - Your day-to-day work/school?	None	3 (3.3)
Mild	0 (0)
Moderate	3
Severe	0 (0)
Extreme or cannot do	35 (35.4)
NA*	58 (58.6)
Participation	S4 - How much of a problem did you have joining in community activities (for example, festivities, religious or other activities) in the same way as anyone else can?	None	13 (13.1)
Mild	1 (1.0)
Moderate	1 (1.0)
Severe	0 (0)
Extreme or cannot do	57 (57.6)
NA*	27 (27.3)
S5 - How much have you been emotionally affected by your health problems?	None	11 (11.2)
Mild	13 (13.1)
Moderate	22 (22.2)
Severe	33 (33.2)
Extreme or cannot do	19 (19.3)
NA*	1 (1.0)

* NA - not applicable, indicating no obvious limitations that would make the attempt of the activity impossible, but the task was not actually tried.

Domain 1 (cognition): 72.8% did not have the opportunity to learn a new task, and 85.9% had no difficulties concentrating on a task for ten minutes.

Domain 2 (mobility): 39.5% of the survivors had no difficulty standing for periods of 30 minutes, while 24.2% had extreme difficulty or could not perform this task. When asked about walking for a kilometer or equivalent distance, 57.6% classified it as difficult, extremely difficult or impossible.

Domain 3 (self-care): 59.7% of the patients had no difficulty washing themselves, and 58.6% had no difficulty getting dressed.

Domain 4 (getting along): 76.7% reported no difficulty dealing with people they did not know, and 80.8% reported no difficulty maintaining a friendship.

Domain 5 (life activities): 33.2% had no difficulty taking care of household responsibilities, but 27.3% reported that this task was difficult or impossible. Regarding performing day-to-day activities in work/school, 35.4% reported extreme difficulty or cannot do, and 58.6% of the survivors could not respond because they were retired or unemployed at the time of hospital admission.

Domain 6 (participation): 57.6% stated that it was difficult, extremely difficult or impossible to join community activities, and 27.3% did not even try to return to those routines. When questioned about how much they had been emotionally affected by their health problems, 22.2% referred to moderate difficulty, 33.2% considered severe difficulty and 19.3% extreme difficulty.

Regarding work/job activities, 27 patients were still on sick leave (27.2%), seven (7.1%) returned to their jobs, 52 (52.5%) were retired before admission to the hospital, nine (9.1%) patients were unemployed before admission to the hospital and still in the same condition, and four (4.1%) patients lost their jobs or were suspended after hospitalization.

## DISCUSSION

One month after hospital discharge after critical COVID-19 leading to admission in intensive care for more than 24 hours, most patients had obstacles that substantially affected their quality of life.

The EQ-5D VAS had a final average rating of 65% on a scale from zero to one hundred, and this information could be used as a quantitative measure of health outcomes, as judged by the individual respondents.^(11)^ The most affected dimension in HRQoL was “usual activities”, which include everyday activities, such as return to work or school or engaging in household tasks and leisure habits. Of the patients, 25% reported moderate, severe or extreme pain or discomfort, and 32.3% reported moderate, severe or extreme anxiety or depression. Carenzo et al.^(15)^ assessed critical COVID-19 survivors 2 months after discharge using the same tool in HRQoL (EQ-5D-5L). Their results were similar to ours, except in the “usual activities” dimension, in which they found a lower percentage of patients with significant handicap.

The health and disability assessment using 12 items WHODAS 2.0 showed that patients had significant problems regarding the following areas: mobility - most not being able to walk for one kilometer and a quarter not being able to stand for half an hour; life activities - less than a half had no difficulties performing household tasks, and only 6% had returned without extreme difficulties in their work/school activities; participation -most could not join community activities and were significantly emotionally affected by their health problems.

No clear consistency was found between the results obtained using each of the two tools. Assessment using 12 items WHODAS 2.0 suggests more severe sequelae than those based on EQ-5D-5L. The reason may be that, 12 items WHODAS 2.0 asks what a person does in a particular domain, while WHOQOL asks what the person feels in that domain.^(12)^ Therefore, survivors stated that they felt better than the disability results using specific and practical questions. According to some publications,^(16)^ real quality of life is usually more consistent with the disability assessment, which patients tend to understate. The patients recognized that they were extremely ill very recently and survived, and that they and their families were prepared for even worse health conditions. Thus, their perception of the actual health state leads to higher classifications of quality of life. Based on the above findings, we recommend the application of both instruments for a complete assessment of the patient´s status.

However, both questionnaires have a pandemic context that influenced participation in activities outside the house and socialization with large groups of people. Therefore, the significant contextual impact on the results obtained must be recognized and stressed. This context makes early identification of PICS among critical COVID-19 survivors even more important, allowing the definition of a care pathway for physical and social rehabilitation.^(5)^ We recognize that 1 month after hospital discharge is too early to actually perceive the sustained impact of critical COVID-19 on the quality of life and disability of the patients. However, this period is likely ideal to identify the main sequelae and define a personalized strategy that could decrease their impact and accelerate rehabilitation and family and social reinsertion. Therefore, what could be valued as a limitation of our study is also, in our opinion, its main strength and originality.

The survivors reported feeling better than the health and disability evaluation results showed, indicating that the self-perception of quality of life is higher than what objective evaluation metrics show. The reason may be due to their expectations. The phone consultations revealed that survivors and their families were prepared for a worse health condition, which may have contributed to their higher classifications of quality of life regarding their actual health state. Thus, we believe that both assessment tools should be applied to maximize the diagnosis of quality of life after critical COVID-19.

The consistency of the population (treated in the same Department of ICM), assessment timing and assessing clinician (the same intensive care nurse with experience in follow-up) are strengths of our study. However, several limitations exist. The evaluation of “return to day-to-day work/school” was often not applied because 58.6% of the survivors were retired or unemployed at the time of hospital admission. The question regarding “learning a new task” also received many “not applicable” responses, likely because of the short follow-up period, justifying the lack of opportunities to learn and develop new tasks. Additionally, the pandemic context influenced the opportunities for participation in outdoor activities and meeting extended family and friends.

## CONCLUSION

This study, the first to assess survivors of critical COVID-19 as early as 1 month after hospital discharge, shows that these patients have significant sequelae from disease and intensive care medicine. Additionally, using more than one standardized and validated quality of life and disability tool helps the assessment and diagnosis of the patient’s status. Finally, an early nursing phone consultation, as part of a holistic follow-up process, may provide counseling and define flows and priorities for medical consultations and specific therapies that may impact rehabilitation and reinsertion.
